# A Scientometric Review of Grain Storage Technology in the Past 15 Years (2007–2022) Based on Knowledge Graph and Visualization

**DOI:** 10.3390/foods11233836

**Published:** 2022-11-28

**Authors:** Guixiang Chen, Jia Hou, Chaosai Liu

**Affiliations:** 1 College of Civil Engineering, Henan University of Technology, Zhengzhou 450001, China; 2 Henan Key Laboratory of Grain Storage Facility and Safety, Zhengzhou 450001, China; 3 Henan International Joint Laboratory of Modern Green Ecological Storage System, Zhengzhou 450001, China

**Keywords:** postharvest losses, food security, grain storage, CiteSpace, visualization

## Abstract

Food storage helps to ensure the food consumption needs of non-agricultural populations and to respond to major natural disasters or other emergencies, and the application of food storage technology can reduce post-harvest food losses. However, there are still obvious shortcomings in coping with large grain losses. Therefore, quantitative analysis of the research hotspots and evolutionary trends of grain storage technology is important to help the development of grain storage technology. This article uses the Web of Science database from 2007 to 2022 as a data sample with the help of CiteSpace software to analyze the basic situation, research hotspots, and evolutionary trends to draw a series of relevant knowledge maps. Visual analysis revealed that the number of publications had grown rapidly since 2015. First, the *Journal of Stored Products Research*, *Journal of Economic Entomology*, and *Journal of Agricultural and Food Chemistry*, with citation frequencies of 929, 536, and 453, should be focused on in order to keep up with the latest research developments in this field. The United States, China, and Brazil occupy dominant positions in relation to grain storage technology studies in general. Purdue University, Kansas State University, and Agricultural Research Institute ranked the top three in terms of the number and centrality of publications. In terms of research hotspots, the centrality of temperature, insects, carbon dioxide, and quality were 0.16, 0.09, 0.08, and 0.08. It shows that the field of grain storage technology in recent years has focused on grain storage temperature, pest control, and grain storage quality research. From the perspective of the evolution trend, the life cycle of emergent words lasts for several years, after which the strength of emergent words slowly decreases and is replaced by new emergent words. Mortality was the first keyword to appear and remained from 2007 to 2011, indicating that research on fumigants and their toxicity, as well as pest mortality under air fumigation and chemical fumigation conditions, became more popular during this period. In recent years, new terms have emerged that had never been used before, such as “grain quality” (2019–2022) and “stability” (2020–2022). We can find that people pursue food quality more with the improvement of people’s living standards. In this context, future research should seek more efficient, safe, economical, and environmentally friendly methods of grain storage and continuously improve the level of scientific grain storage.

## 1. Introduction

Food is the basic means of living for the survival and development of human society and is an important material to stabilize the market and ensure the livelihood of the nation [1]. Food security is an important guarantee for world peace and development, as well as an important foundation for building a community of human destiny, and all countries in the world give top priority to the issue of food security [2]. However, global food security is facing serious challenges. In recent years, adverse factors affecting world food security have increased [3]. The interplay of economic recession and food trade supply chain disruption is caused by international conflicts and extreme weather [4]. COVID-19 has exacerbated instability and uncertainty in the global food supply system [5]. The new crown outbreak in late 2019 not only poses a formidable challenge to global health security but also puts global food security in jeopardy [6]. Food insecurity is primarily related to food waste and food loss; food waste is the reduction in the quantity or quality of food due to the decisions and actions of food service providers and consumers [7]. Food losses include the harvested crop throughout the entire food supply chain. Losses can be broadly classified as weight loss, quality loss, nutritional loss, seed viability loss, and commercial loss [8]. Grain is a living organism with hygroscopic and respiratory characteristics. Grain respiration during the storage chain causes loss of nutritional value, metabolites, and physiological parameters (germination and vitality) of food products, while the development of fungal and insect pests inside the grain pile directly accelerates the process of grain deterioration [9]. Appropriate measures are needed to maintain the quality of grains and reduce grain losses [10]. Solutions to the global food crisis and improvements in the food security situation can be achieved by increasing food production and preventing losses in the food supply chain [11]. So far, most scientific research efforts have been aimed at increasing agricultural crop yields, but reducing post-harvest losses may be a sustainable solution for increasing food availability, reducing pressure on natural resources, eliminating hunger, and improving farmers’ livelihoods, especially in developing countries [12,13,14].

The current literature that systematically summarizes the current status of grain storage technology research is mostly a qualitative summary and collation analysis. Manandhar et al. [15] evaluated different post-harvest grain storage practices of farmers in developing countries around the world and different post-harvest losses associated with the grain supply chain, based on which a rational grain storage structure was proposed and used to effectively reduce post-harvest grain storage losses. Hussain et al. [16] reviewed the recent research progress in the application of classical and non-destructive techniques to evaluate the safety and quality of cereals and their products. The advantages and limitations of these technologies are presented, and future trends and challenges are elucidated. Paul et al. [17] reviewed the current status of established post-harvest pest control methods and emerging pest control technologies and discussed the role model and potential of these technologies in developing a green approach for effective control of storage pests at all stages. Moirangthem et al. [18] reviewed the latest applications of ionizing radiation, modified atmosphere, and dielectric heating in grain storage pest control. Reading review articles in the field of grain storage technology reveals that although a large number of papers and research literature have been published in different journals by scholars in the field of grain storage technology. However, there are very few systematic analyses using new bibliometric tools or visual analysis tools (e.g., CiteSpace). There is also less review literature that combines quantitative visualization methods to analyze the current state of research and future research trends in the field.

It is necessary to conduct a systematic analysis of existing research results to gain a deeper understanding of the current status and future trends of grain storage technology research. Based on this, this paper conducted basic situation analysis, research hotspot analysis, basic knowledge identification, and evolution trend analysis using CiteSpace software and Web of Science database from 2007 to 2022 related literature in the field of grain storage technology as data samples. The purpose of the work was to show the evolution path of grain storage technology research by drawing a series of related knowledge maps. It provides a reference for further theoretical research and practical exploration of grain storage technology.

## 2. Data Collection and Research Methods

### 2.1. Data Collection

The research object of this paper is the literature related to the field of grain storage technology, and the data source is the Web of Science (WOS) database. Web of Science is the world’s largest comprehensive academic information resource covering the largest number of disciplines, with over 9000 peer-reviewed, high-quality journals (covering 178 disciplines) in the natural sciences, engineering, biomedicine, and other research fields [19]. The rich and powerful search function of the Web of Science makes it easy to quickly find valuable scientific information and obtain a comprehensive understanding of research information on a particular subject or topic. Users can search across all databases subscribed to the platform at the same time or select one of them for a single search [20]. The Impact Factor (IF) introduced by Web of Science has become a common international journal evaluation index, which is not only a measure of the usefulness and display of the journal but also an important indicator of the academic level of the journal and even the quality of the paper [21]. CiteSpace is based on the WOS data format, and data downloaded from a non-WOS database must be converted to the WOS data format. A subject search was selected to cover the research status in the field of grain storage technology as comprehensively as possible, and the literature data spanning about 15 years from January 2007 to May 2022 were collected with a cut-off time of 31 May 2022. The search topics were “Grain Storage Technology, Low-Temperature Storage, Controlled Atmosphere Storage, Grain Storage Technology AND Ventilation, Refrigeration, Atmosphere, Carbon Dioxide, Nitrogen, Gas, Temperature, Humidity, Insect, hermetic, detection, inspect, granary”. A total of 1645 literature data were collected.

### 2.2. Research Methods

Knowledge mapping combines co-citation analysis theory and pathfinding network algorithms by integrating modern bibliometrics and information science. Present the development history, frontier areas, and research hotspots of this research topic in the form of a visual map [19]. It is difficult to sort out and judge the literature in a certain research field as a whole in the traditional literature analysis method. The traditional literature analysis method belongs to qualitative analysis, which is by sorting out the main contents of the literature, and it is difficult to grasp the research status comprehensively and accurately from a large amount of data. It depends on the size of the researcher’s reading volume and summarizing ability, which has a certain subjectivity [22]. The quantitative analysis represented by knowledge mapping has begun to be widely used in review research with the rise and popularity of Internet technology, which reveals the dynamic development pattern of the knowledge domain through data mining, information processing, knowledge measurement, graphic drawing, and provides practical and valuable references for disciplinary research [23]. At present, the main tools for mapping knowledge in academia are CiteSpace, SPSS, Ucinet, VOSviewer, etc., among which CiteSpace software is the most commonly used tool. The main function of CiteSpace software is to present and analyze the evolutionary trends and knowledge association status of disciplinary frontiers through visualization functions such as keyword cooperation, institutional distribution, author collaboration, and literature coupling [24]. The information visualization tool used in this paper is CiteSpace software, version CiteSpace 6.1.R1 (64-bit). CiteSpace is a scientific bibliometric visualization and analysis software developed by Prof. Chaomei Chen of Drexel University, based on the Java environment platform, which shows the research field through the size of nodes, network connectivity, and other elements CiteSpace is a scientific bibliometric visualization and analysis software based on Java environment platform [25]. Centrality is a measure of the importance of a node in a network by which the importance of the literature is discovered and measured, according to CiteSpace author Dr. Chen [26]. Given this, this paper uses CiteSpace software as a visualization tool for the study to draw a series of relevant knowledge maps and analyze the research overview and research dynamics in the field of grain storage technology.

The process of conducting the review in this paper is divided into three steps: in the first step, the current state of research in the field is analyzed based on the number of publications in the literature. The authors and institutions are analyzed using CiteSpace, and the main research in the field is analyzed by studying representative literature. The second step consists of an analysis of analyzing the research hotspots, frontiers, and trends in the field. CiteSpace is used to perform co-word analysis and cluster analysis on the literature and then study representative literature based on the results to derive research hotspots, frontiers, and trends. In the third step, future research directions are proposed by synthesizing the results of the analysis. 

## 3. Results

### 3.1. Trend Analysis of Literature Publication

**Figure 1:**
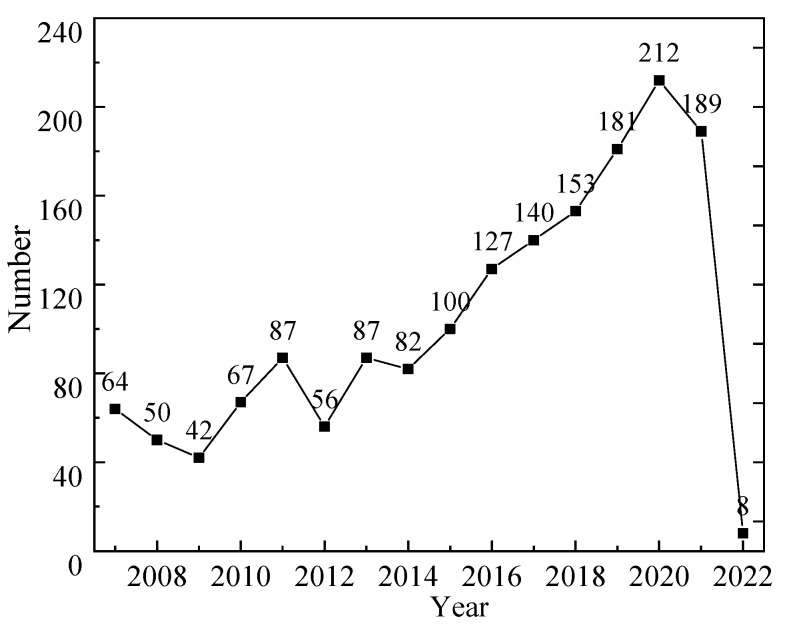
The number of articles published from 2007 to 2022.

The annual number of publications and the annual cumulative number of publications for grain storage technology research in the Web of Science database was plotted, as shown in Figure 1.

The trend of publications on the Web of Science from 2007 to 2015 is relatively stable. The increasing trend is slow, with an annual average of about 60 publications, although the cumulative number of publications has increased each year. It belongs to the budding stage of research discussion and accumulation, and it has not yet formed a more complete disciplinary form. The increase in the number of publications accelerated from 2016, reaching 153 in 2019 and peaking at 212 in 2020. This indicates that more and more people are engaged in research on grain storage technology, the heat of research on grain storage technology is increasing, and scholars are paying more attention to it. The decline in the number of published papers by 2021 may be related to the emergence of emerging fields. It may take some time for the research to produce results as technology evolves and scholars begin to combine research with smart technologies. The 2022 article is not representative of the whole year because it was only retrieved in May.

### 3.2. Analysis of Journal Co-Citation Network

**Figure 2:**
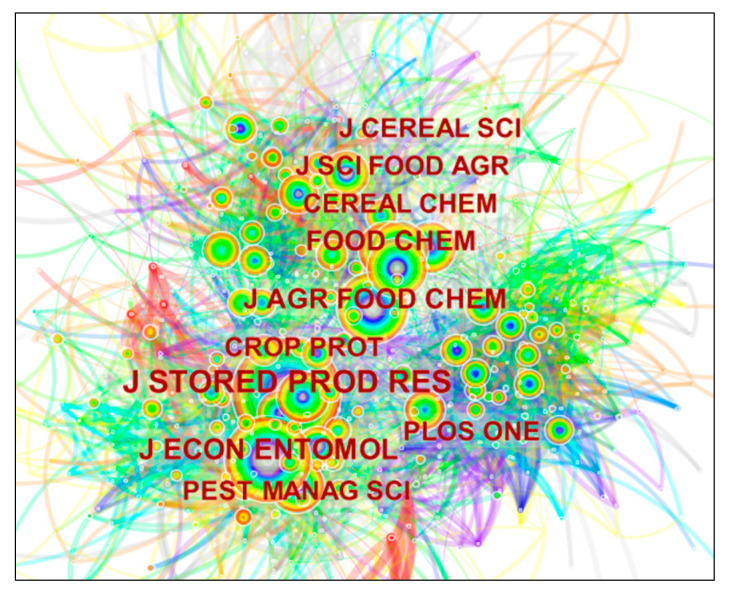
Journal co-citation network.

Journal co-citation analysis is performed on journals in which cited articles appear (Cited Journal), and the co-citation network of journals can be plotted by CiteSpace. The value of “Year Per Slice” is set to 1, a time partition of 1 year is used, and the node type is set to Cited Journal, which finally generates a knowledge graph of grain storage technology research co-cited journals, as shown in Figure 2. Table 1 shows the top 15 cited journals.

Figure 2 shows the results of the co-citation analysis of journals published in the literature related to grain storage technology. At the same time, the level of influence of each journal publication can be visualized by analyzing the citation frequency and centrality of journals [23]. To comprehensively reflect the distribution pattern of co-cited source journals, the top 10 journals in terms of citation frequency and their centrality were summarized by querying the relevant information in the background of the atlas. The top three journals with the most nodes, as seen in Figure 2, are Storage Products Research, Economic Entomology, and Agricultural and Food Chemistry, with citation frequencies of 929, 536, and 453, respectively. It shows that these three journals ranked top in citation frequency which are important windows to explore the research progress and development trend in this field. Centrality is a measure of the importance of nodes in the network by which the importance of journals is found and measured [27]. *Journal of Cereal Science*, *PloS One*, and *Journal of Agricultural and Food Chemistry* are the top three journals in terms of centrality. It can be found that there is no positive correlation between citation frequency and centrality by comparing journal frequency with centrality ranking, which means that even a high citation frequency does not necessarily indicate that the journal is influential. The *Journal of Agricultural and Food Chemistry* is the most cited and centered journal. This indicates that it has the greatest utility in the overall network of cited journals and is the top journal in the discipline of agricultural and forestry sciences. The *Journal of Cereal Science* and the *Journal of Food Science* is not among the top journals in terms of citation frequency, but their centrality ranks first and second among all journals, and their importance and authority in research on grain storage technology cannot be overstated. The *Journal of Cereal Science* was established in 1983 to provide an international forum for the publication of high-level original research papers on the functional and nutritional quality of cereals and the relationship of their products to the grains used. The *Journal of Food Science*, published in 1961, covers all aspects of food science.

The journal co-citation analysis shows the research journals that cite journals related to grain storage technology in international studies. It indicates that the research results of these journals are recognized and adopted in international studies, and the results can guide researchers to quickly find suitable journals for publishing papers related to grain storage technology.

### 3.3. Analysis of Author Cooperation Network

**Figure 3:**
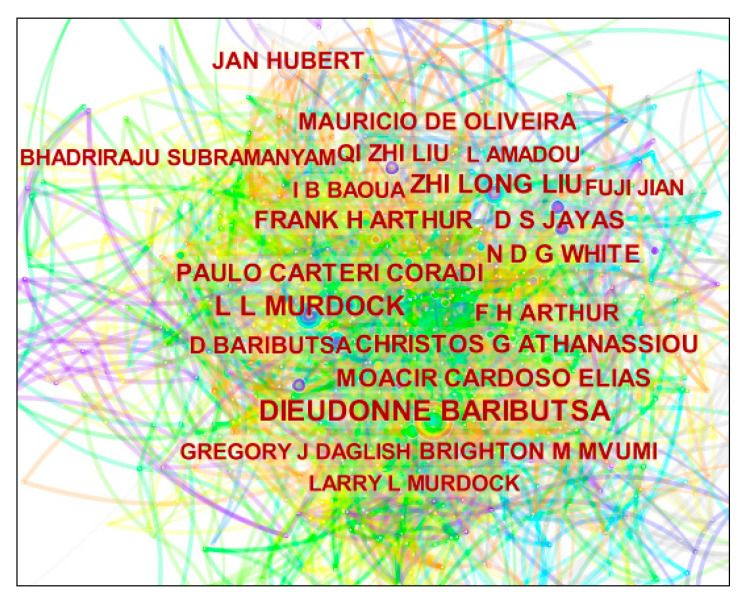
Author cooperation network map.

The analysis of posting authors and their collaborative networks can identify the main research teams in a research field, present the collaborative relationships between different researchers, and clarify the core figures in the field [27]. Based on the CiteSpace visualization tool, the network cooperation analysis was performed by clicking the “Author” function in CiteSpace, and the lines between the nodes represented the cooperation status among the cited authors in the same literature. The collaborative network knowledge graph of authors posting in the field of grain storage technology is shown in Figure 3, with the number of co-occurring nodes being 565, the number of connections being 653, and the network density being 0.011. The top 20 authors posting according to the final algorithm of CiteSpace are listed in Table 2.

Consistent with observations in other research areas, a small group of prolific authors has contributed considerably to research publications on grain storage technology. The most productive author in grain storage technology research is Baributsa from Purdue University, with research interests in Pest Management, Agricultural Entomology, Storage Entomology, and Grain storage. Murdock, also from Purdue University, has the same research interests as Baributsa, followed by Arthur from Agricultural Research Service United States. He interconnects Ecology, Warehouse, Biopesticide, Physical control, and Relative humidity in the investigation of issues within PEST analysis. His Pesticide study incorporates themes from Pest control, Chemical control, and Integrated pest management. Authors with a high volume of publications are not necessarily highly centric-scholars. They are the leading scholars who have had a fundamental impact on the development and evolution of grain storage technology when the nodes have high school cardinality [28]. Their work deserves more attention because it may change the direction of grain storage technology research. In terms of the influence of scholars, Liu and Baoua have fewer publications but higher centrality. The strength of representative scholars and core research teams in the field of grain storage technology can be determined by analyzing the collaborative networks of the authors. Authors who published a large number of articles showed clear network characteristics, with Baributsa and Murdock forming the largest core collaborative network. This indicates that these core authors have established a high-yield author research team in the field of grain storage technology, which has initially taken shape. It can be noted that the density of graphical linkages is particularly high, which proves that the research on grain storage technology has initially matured and formed a good scale of clustering and made more contributions to the development of grain storage technology. The fragmented scholars should cooperate and communicate more with other scholars to work together for the long-term development of the field of grain storage technology.

### 3.4. Analysis of Country Cooperation Network

**Figure 4:**
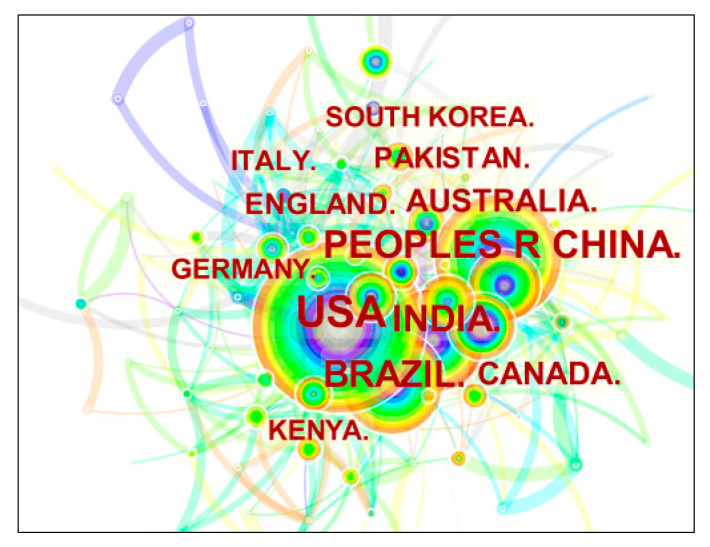
Country cooperation network map.

Based on the CiteSpace visualization tool, that node type selects the Country node and runs the software to obtain the country (region) cooperation map. The cooperation network knowledge map of research literature issuing authors in the field of grain storage technology is shown in Figure 4, where the number of nodes is 565, the number of connections is 653, and the network density is 0.011. The number of issuing in the top 20 countries is listed in Table 3 according to the final algorithm of CiteSpace.

The three countries with the largest nodes are the United States, China, and Brazil, with 354, 243, and 173 publications, respectively. It is relatively one-sided to judge the development degree of grain storage technology in each country only from the number of published. Therefore, it can be found that the United States is the most influential country in the field of grain storage technology by comparing Figure 4 and Table 3. The U.S. leads other countries in both frequencies of publication and centrality and is the most active country in conducting research. It shows that the United States has the closest cooperation and exchange with other countries and plays an important role in international cooperative research. China and Brazil rank second and third, respectively, in terms of the number of publications but are slightly behind the UK in terms of centrality. China and Brazil still need to strengthen the links and cooperation with other countries to improve the relevant technology and enhance international influence to promote the further development of grain storage technology. The low number of published articles but high centrality rankings in the UK and Spain indicate that these two countries are also among the world leaders in the field of grain storage technology and still have room for development.

### 3.5. Analysis of Institutional Cooperation Network

**Figure 5:**
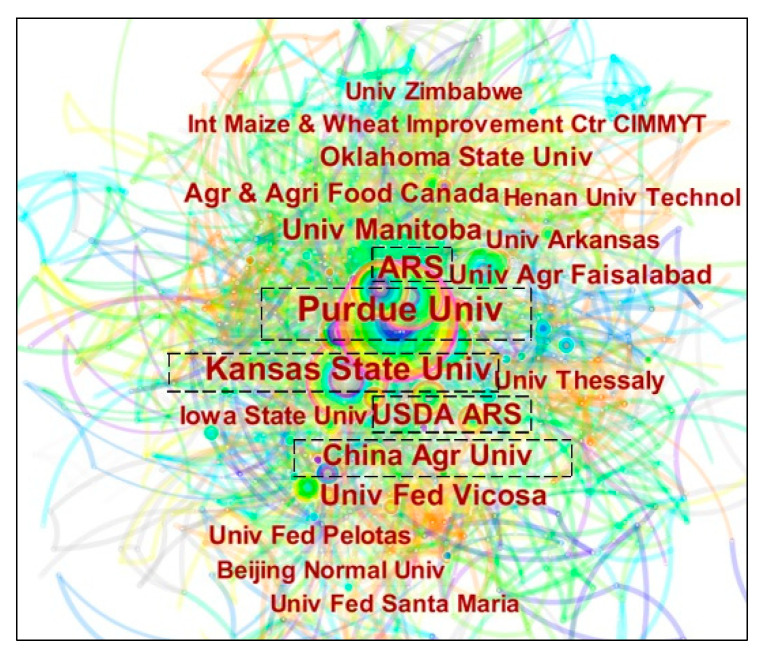
Institutional cooperation network map.

The network cooperation analysis of institutes and institutions can be performed by clicking on the “Institution” function in CiteSpace. The collaborative network knowledge graph of research institutions in the field of grain storage technology is shown in Figure 5, with the number of cooperation nodes of 128 research papers between 2014 and 2021 being 1743 and the number of links at 3476, 1743 and 3476. n in top-n is set to 60 (meaning that the 60 most frequently cited documents are extracted in each time slice. The top 20 major research institutions with published articles according to the final algorithm of CiteSpace are listed in Table 4. 

From Figure 5, it can be seen that a mature cooperation network has been formed among international research institutions. The number of connected lines shows that international research institutions on grain storage technology have close co-citation relationships and considerable prospects for subsequent development. The top five institutions in terms of the number of publications were Purdue University (86), Kansas State University (55), Agricultural Research Institute (51), USDA Agricultural Research Service (41), and China Agricultural University (39 times), while the top five institutions in terms of centrality were Purdue University (0.23), Kansas State University (0.17), Agricultural Research Institute (0.14), University of Agriculture Faisalabad (0.12), and Iowa State University (0.09). Among them, Purdue University, Kansas State University, and Agricultural Research Institute ranked the top three in terms of the number and centrality of publications. It proves that these three institutions have a high influence in the field of grain storage technology and have strongly promoted the development of grain storage technology research. It can also be seen from Figure 5 that the largest inter-team collaborative network was formed with Baributsa as the leader, and the network consisted of 86 writers, including Murdock from Purdue university and Njoroge from the University of Florida. The research team led by Baributsa from Purdue university has become a stalwart in the field of grain storage technology. Cooperation between universities, enterprises, research institutes, and other research institutions can better enable researchers to understand real-life needs, inspire research and form new research directions. Therefore, universities should strengthen cooperation and exchange with enterprises and research institutes in future research and work together for the long-term development in the field of grain storage technology.

### 3.6. Analysis of Hot Research Topics

**Figure 6:**
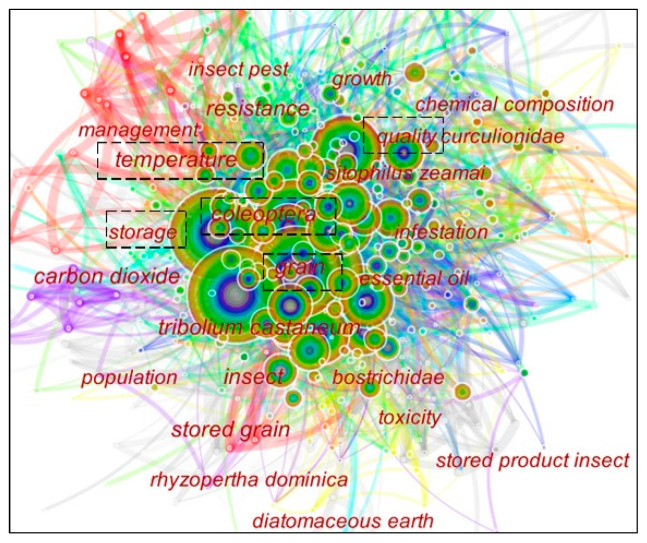
Keyword cooperation network map.

CiteSpace‘s cooperation analysis of keywords and nomenclature in the literature provides access to the hotspots of grain storage technology research [27]. The keyword cooperation analysis was performed by selecting the “Keyword” function item to obtain a cooperation map of high-frequency keywords and terms, as shown in Figure 6, containing 491 nodes and 3811 connections. The circles in Figure 6 represent keywords and Table 5 lists the keywords with a cooccurrence frequency greater than 20 times.

The cooperation frequency of grain and storage in the keywords as the retrieved subject terms are 196 and 191, respectively, which rank first and second in the cooperation frequency of keywords. The centrality of temperature, insect, carbon dioxide, and quality were 0.16, 0.09, 0.08, and 0.08, respectively, ranking in the top four of centrality. It can be inferred that the research on grain storage technology is mainly focused on the control of grain storage temperature and humidity, pest control, and grain quality. The keywords related to grain storage pests have high citation frequency. Finally, the following three major research hotspots in grain storage technology are derived by analyzing the frequency and centrality of each key subdivision in the chart:

1. Temperature: In the grain storage ecosystem, the temperature of the grain pile rises abnormally due to the concentration of heat, or the phenomenon that the temperature of the grain should rise instead of falling is called grain pile fever. It will further develop into mildew and eventually affect the use value and edible value [29]. Grain heat is mainly the result of respiration and heat accumulation by organisms in the grain pile. Reed et al. [30] studied the response of storage molds to different initial moisture contents of maize stored at 25.1 °C and its effect on respiration rate and nutrient composition. Aldred et al. [31] investigated the effect of three essential oils and antioxidants on the control of growth and ochratoxin production by Penicillium wolframite and Aspergillus accidentally under different moisture and temperature conditions. Preventing grain storage heat needs to do a good job of insulation and moisture, improving storage conditions, timely ventilation, and airtight. Doing a good job of predicting and forecasting grain fever, early detection of problems, and timely treatment is also an important job to prevent losses due to grain storage fever [29]. In addition to simple indicators of anomalous changes in grain temperature and moisture [32], it is possible to predict the heat in grain storage by measuring the evolution of microbial taxa in grain storage [33]. Of course, it also needs to be equipped with appropriate equipment and trained inspectors. For the treatment of fever, different measures should be taken according to the cause of the fever. The most fundamental measure is to dry treatment if the grain heat and mold growth are caused by wet grain, such as drying, drying or mechanical ventilation, water, and temperature reduction.
2. Insect: The respiration of the stored grain pests will change the moisture and temperature of the grain pile, which will affect the grain security, cause weight loss and seriously decrease the quality. The excreta and carcasses of stored grain pests can contaminate food, leading to substandard health indicators and affecting human health [33]. Gas-conditioned grain storage is the world’s most recognized green, safe, and effective grain storage pest control technology, unlike the traditional drug fumigation to kill insects, which is filled with a high concentration of carbon dioxide or nitrogen gas in a well-sealed silo to destroy the living environment of insects and mold. This results in the death of pests and reduces the respiration of grain to improve grain quality and safe storage [34]. In addition, a portion of scholars has studied low-risk, less contaminated fumigant insecticides. Hertlein et al. [35] concluded that carbendazim is effective in controlling important pests associated with grain storage, as well as insect strains that have developed resistance to other grain protectants and have low toxicity to mammals. Safe storage and protection of grain also include the use of plant-based fumigants as a green control technique. Several scholars have studied the repellent effect and fumigant activity of herbs and essential oils against storage pests [36,37,38]. A theoretical basis for the development and application of plant fumigants in the integrated management of food pests. More research is needed in the future to develop formulations to improve their efficacy and stability and reduce their cost. Further experiments are needed to ensure that the consumption of fumigated grain does not negatively affect humans and other animals. There are also biological and physical control methods. Temperature management is one of the best biological control methods, which involves ventilating and cooling the grain to inhibit the growth of insect populations, as well as using thermally forced air distributed in food processing facilities to thermally kill insects. Nanopreparations also have great potential in developing alternative pest control methods. Rajkumar et al. [39] showed that polymeric chitosan nanoparticles could improve the insecticidal activity of essential oils by controlling the effective release of essential oils to storage product pests. Physical control methods have the characteristics of safety and are not easy to produce physiological resistance, which can effectively prevent and control grain storage pests and achieve the purpose of safe grain storage. Inert powders have all had a long history of use as grain storage protectants, and recent studies have shown that diatomaceous earth is considered the best class of natural powder insecticides available. The inert powder has a long history as a protective agent for stored grain. Recent studies have shown that diatomite is considered to be the best among natural powder insecticides [40]. Erturk et al. [34] studied the insecticidal activity of a new diatomaceous earth wettable powder against rice weevil. It was also tested for its effectiveness against adult Streptococcus Ricinus under laboratory conditions. Sealed storage has been of interest as a physical method to control post-harvest pests, and there is a growing body of research on the use of sealed containers to control stored pests. Njoroge et al. [41] used O_2_ sensors, acoustic sensors, and visual observation to further measure the effect of confined storage on pest activity and mortality. Abass et al. [42] tested seven maize storage methods based on the Central Corridor maize growing system in Tanzania and compared them with the traditional polypropylene bag storage method. The results showed good results for the insecticidal treatment of maize using the traditional Tanzanian method of storage in polypropylene bags. Chemical pesticides should be avoided for public health as well as health reasons. Therefore, closed storage without pesticides is preferred, but storage materials need to be made affordable to farmers. It is also important to ensure that farmers handle and manage these technologies properly. Grains must be properly dried before storage, and re-infestation during the intermittent opening of sealed containers should be prevented as much as possible.
3. Quality: The results showed good results in the insecticide treatment of maize using the traditional Tanzanian method of storage in polypropylene bags [43]. Post-harvest grain will continue to respire during storage and produce microorganisms, such as mold, that can be harmful to the quality of stored grain. Temperature, air humidity, and time are the main factors causing changes in the grain storage process. High temperatures and humidity can lead to deterioration in grain storage quality and production losses. Qu et al. [44] investigated the effect of microwave heating of wheat seeds on flour gluten, flour quality, pasting properties, and baking (buns and cookies) properties. The experimental results showed that microwave treatment could inactivate LA and LOX and prolong the storage period. Keskin et al. [45] evaluated the effect of wheat sample storage and granaries L. infestation on the process characteristics of wheat samples, and the results showed that the physical, chemical, and physicochemical properties of wheat and flour were affected by wheat and flour mold. Mutungi et al. [46] conducted on-farm experiments to investigate the effects of smallholder farmers’ maize harvesting and handling practices on the quality of products before and during storage at two different agricultural sites. A rapid method to identify and measure stored grain quality is needed that can help reduce stored grain quality losses and establish appropriate storage conditions to verify how storage technology affects the rate of quality deterioration. Near-infrared spectroscopy (NIRS) is an efficient technique for the chemical characterization and screening of agricultural crops. Belzoni et al. [47] explored the potential of near-infrared spectroscopy (NIRS) as a process analytical technology for the evaluation of soybean quality under different storage conditions. Pohndorf et al. [48] used kinetic models and Arrhenius’ law to verify the oxidative stability of soybeans under different storage conditions. The study also provided technical support for controlling temperature and relative humidity during soybean storage in hot and humid regions. A large number of studies have shown that gas conditioning and low-temperature storage as a green grain storage technology can effectively solve the problem of residual harmful substances in stored grain and slow down the aging of stored grain, and effectively inhibit grain quality deterioration. However, the ultimate purpose of the storage is circulation. Therefore, the quality change of paddy after the storage is directly related to the economic efficiency of grain enterprises, and the research on controlling the quality change of paddy after unsealing is also extremely important.

### 3.7. Analysis of Frontiers Trending

**Figure 7:**
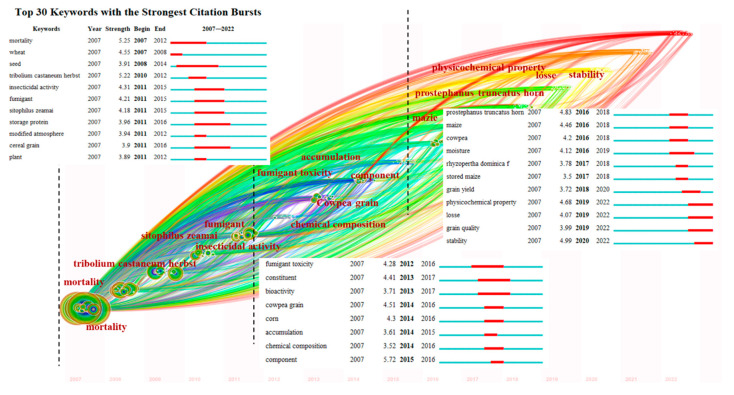
Top 30 burst keywords detection.

In this paper, the Kleinberg burst detection algorithm was used to detect burst words in the literature space and identify several terms that represent the frontiers of research [27]. The node type was set to “Keyword” and “Timezone”, and the top 30 emergent words were arranged in ascending order of their emergence time together with the keyword time zone map, as shown in Figure 7. Year in the figure represents the year of the first occurrence, Strength represents the burst intensity, and Begin and End represent the years of beginning and ending within the burst period.

As shown in the figure, the life cycle of emergent words lasts for several years, after which the strength of emergent words slowly decreases and is replaced by new emergent words. In this way, it is possible to obtain a quick overview of the overall leap and transition process of hot topics in the field of grain storage technology. Mortality was the first keyword that started to emerge and was maintained from 2007 until 2011, during which mortality (2007–2012), tribolium castaneum herbst (2010–2012), insecticidal activity (2011–2015), fumigant (2011–2015), and modified atmosphere (2011–2012) all had high emergence intensity. This was closely followed by storage protein (2011–2016), sitophilus zeamai (2011–2015), wheat (2007–2008), and seed (2008–2014). It shows that studies on fumigants and their toxicity, as well as studies on pest mortality under chemical fumigation conditions, were more popular during this period. Overall, the current study is relatively homogeneous. Fewer emergent words appeared in 2012–2015, which were fumigant toxicity (2012–2016), constituent (2013–2017), bioactivity (2013–2017), cowpea grain (2014–2016), corn (2014–2016), accumulation (2014–2015), chemical composition (2014–2016), and component (2015–2016), with cowpea grain and component being more prominent and becoming the main research in this phase. In addition, fumigation and chemical pest control still showed research fervor. The years 2016–2020, on the other hand, showed a large number of emergent words, with stability (2020–2022), prostephanus truncatus horn (2016–2018), physicochemical property (2019–2022) with the highest centrality of 4.99, 4.83, and 4.68 respectively. There are also emergent words, such as grain quality (2019–2022) and stability (2020–2022), that have never appeared in the past. It is not difficult to find that the research frontiers of grain storage technology research in recent years are fumigant toxicity, physicochemical property, and grain quality. People are seeking foods with improved flavor while limiting the use of additives as people’s living standards improve [49]. Weight loss, quality loss, nutrient loss, and seed vigor loss have become new research hotspots. In this context, achieving high-quality and safe food and identifying appropriate processing and sterilization technologies have also become key issues. Several food processing techniques have been explored and implemented with the ultimate goal of maintaining the safety, freshness, and nutritional attributes of the food. It indicates that the research related to grain storage technology will be carried out around it in multiple aspects and dimensions for quite some time in the future. Protecting food from loss is the greatest principle of food storage and the goal of maximizing the benefits to all and society. Currently, the protection of stored grain has been introduced and is gradually becoming intelligent [32]. It can achieve low temperature, low oxygen, and low energy consumption green management of the grain in stock to achieve loss reduction, consumption reduction, and freshness preservation with the Internet of Things + intelligent grain storage technology [50]. Food damage can even be reduced to near zero if the technology is mature and scientific storage and management practices are used [51].

## 4. Discussion

The summary of the above results found that the relevant scholars have conducted more in-depth research on grain storage technology and achieved certain scientific results. Further reading of the relevant literature summarizing the important nodes involved in the operation of the software revealed that the following technologies are mainly used to prevent grain storage losses: grain storage pest control technology, low-temperature storage technology, and airtight and air-conditioned grain storage technology. The quality of the grain before storage is also an important factor in determining whether it can be stored safely in the long term. Therefore, the grain can be treated with UV-C, microwave, magnetic field, and other physical methods before storage [52].

Further analysis revealed that pest control is a key aspect of grain storage to ensure grain quality, as losses during grain storage are mainly caused by pests [18,53]. At present, there are three main methods of grain storage pest control: biological, physical, and chemical. Physical control techniques for grain storage pests include high-temperature and low-temperature insecticides, air conditioning (high CO_2_ or low O_2_) insecticides, radiation, inert powder, drying, etc. The high-temperature insecticide has the advantages of short treatment time and good effect, but it is only suitable for the insecticidal treatment of a small amount of food, such as farmers [54]. Low-temperature freezing to kill insects is due to the poor cold tolerance of grain storage pests [55]. Mechanical ventilation can be used to reduce the temperature in practice and do a good job in the warehouse insulation transformation, so the cryogenic freezing insecticide and cryogenic storage technology have an organic combination. The control of grain storage pests through drying technology includes two aspects, one is synchronized with high-temperature insecticide technology [56], and the other is by reducing moisture [57]. The growth and development of grain storage pests are inhibited under the ecological conditions of dry grain piles. However, the control of stored grain pests may cause the weight loss of stored grain along with water loss of stored grain, which will bring huge economic losses to the grain storage enterprises [54]. Radiological control is the direct killing of pests or making them sterile and unable to reproduce by treating grains with high-energy radiation using radioisotopes or electron gas pedals [58]. The history of using inert powder as a grain storage protectant is very long. There are four basic types of inert powder used as grain storage protectants: first, clay, sand, and dust; second, synthetic silica-oxygen gel; third, non-oxidized silica-like powder; and fourth, diatomaceous earth [59]. Among them, diatomaceous earth may have a good prospect as a grain storage protective agent. The main advantage is that it has very low toxicity to higher animals, and diatomaceous earth is produced in more than 30 countries around the world [60]. In the past 10 years, relevant scholars focused on natural enemies of pests, entomopathogenic microorganisms, insect growth regulators, research and application of insect pheromones, and other aspects. The United States and other developed countries attach great importance to the research and development of biological insecticides or pest control agents, and many products are already on the market [61]. The application of monitoring traps will greatly improve the safety of grain storage if high concentrations of natural pheromones are extracted or efficient pheromone analogs of grain storage pests are synthesized based on controlled production costs for pheromone control of grain storage pests [62]. Chinese medicinal plants are rich in sources and species, and many of them have insecticidal activities. It is feasible to study the insecticidal effects of herbal plants and their extracts and use them to develop grain storage-related agents [63]. With the in-depth research and application of relevant advanced results, the innovation in biological control of grain storage pests focuses on the research of large-scale cultivation and release techniques of natural enemies of pests [64]. The essence of pest management is to take full advantage of environmental and natural factors to control pests. Biological and physical methods are considered first, and chemical methods should be used only when control fails. Chemical control methods consist of the use of insecticides to control pests in grain storage. The advantages are rapid and thorough insecticidal effects and low treatment costs. The disadvantages are contamination of the grain and the toxicity of the insecticide to both humans and animals [65]. It is still the most cost-effective means of killing insects despite the many disadvantages of chemical control methods.

Low-temperature food storage technology will receive more and more attention as mankind faces serious challenges to survival and the environment [66]. Several countries have applied low-temperature grain storage technology, which has greatly reduced the amount and use of grain storage chemicals. So far, the main way to achieve low temperature is still the grain cooler and other mechanical refrigeration, ventilation, underground low temperature, and solar adsorption refrigeration [67]. Among them, mechanical ventilation is the most commonly used low-temperature grain storage technology, which can effectively reduce grain temperature, eliminate condensation, reduce grain storage moisture, reduce moisture gradient, adjust the quality and increase humidity, and eliminate odor from grain piles [68]. It is commonly used in Australia, the United States, Canada, and other developed countries, where most farm vertical silos are equipped with mechanical ventilation facilities. From the perspective of international research and development dynamics, the focus of technological innovation in the coming period is to research and develop automatic control systems for grain storage ventilation and cooling with artificial intelligence [67]. The main purpose of air conditioning grain storage technology is to kill pests by changing the concentration of O_2_, N_2_, and CO_2_ in the air [69]. Although the cost of fumigation is higher than the general fumigation technology, it can effectively maintain the grain quality without any pollution of the stored grain and does not affect human health [70]. The key to its technological innovation is the development of economic and practical airtight technology and airtight materials for grain silos, which can effectively improve the airtightness of grain silos and reduce the cost of grain storage while significantly reducing the cost of silo construction [71]. As a mature green grain storage technology, air conditioning and airtight grain storage can effectively maintain grain quality, effectively replace chemical supplies, and have no pollution to grain storage [72].

In summary, relevant scholars have conducted more in-depth research on grain storage technology and achieved certain scientific results. The research conclusions and recommendations made through the analysis of knowledge mapping are as follows. From the research profile, more and more people are engaged in grain storage technology research, and the heat of grain storage technology research is increasing. Fragmented scholars should cooperate and communicate more with other scholars to work together for the long-term development of the field of grain storage technology. In terms of research hotspots, the field of grain storage technology in recent years has focused on grain storage temperature, pest control, and grain storage quality. How to implement temperature-controlled grain storage technology in different bin types, grain storage ecological zones, and different grain varieties are the focus of researchers. The use of appropriate control techniques is important to maintain grain quality and quantity because grain storage pests cause significant grain losses each year. China is rich in sources and species of medicinal plants, and with the in-depth research and application of relevant advanced achievements. Various special active substances can be extracted and synthesized by using high technology and precision instruments in the future and used in combination with various existing means such as gas conditioning and fumigation. From the evolutionary trend, intelligent insecticidal instruments such as IoT insecticidal lamps are gradually coming to the fore. Pest data should be systematically collected and used for pest management decisions with the deepening of research on storage grain pest control and new tools for studying insect population sampling and the application of these data in computer-aided decision-making. Combining grain storage technology with IoT technologies, wireless transmission technologies, and big data analytics to maximize their advantages also deserves further study. In summary, relevant scholars have conducted more in-depth research on grain storage technology and achieved certain scientific results.

## 5. Summary

This paper uses CiteSpace to conduct quantitative and qualitative research in the field of science in a visual manner to improve knowledge and understanding of the research field of grain storage technology. The quantitative visualization and qualitative analysis were conducted using the Web of Science database from 2007 to 2022 as the data sample. It provides a valuable reference for a better and faster understanding of the basic overview and research progress in the field of grain storage technology research. It helps scholars broaden their research horizons, identify future research directions, and provide references for future research of related scholars. Currently, grain storage technology has introduced high-tech equipment and is gradually becoming intelligent. It is possible to reduce food damage to even close to zero using scientific storage and management practices. The future should seek more “efficient, safe, economic, health, green, environmental protection”, new methods of grain storage, and new ways to continuously improve the level of scientific grain storage and improve social and economic benefits. Scholars should try more interdisciplinary collaborative research in subsequent research work to further advance the development of grain storage technology research. However, this study has some limitations. Although a relatively comprehensive literature database has been established, the choice of the search strategy and manual screening limited the collection of literature, and the data set not included in this study resulted in a small sample of missing data, which would affect the precision of the scientometric and thematic analysis to some extent. Based on these limitations, a more in-depth content interpretation and analysis are recommended for future studies.

## Figures and Tables

**Table 1:** Journals information table of the top 15 cited frequency.

Ranker	Cited Frequency	Centrality	Journal
1	929	0.01	*Journal of Stored Products Research*
2	536	0.01	*Journal of Economic Entomology*
3	453	0.03	*Journal of Agricultural and Food Chemistry*
4	349	0.03	*Food Chemistry*
5	318	0.02	*Crop Protection*
6	311	0.03	*Cereal Chemistry*
7	288	0.02	*Journal of The Science of Food and Agriculture*
8	256	0.02	*Pest Management Science*
9	249	0.06	*Journal of Cereal Science*
10	247	0.04	*PloS One*
11	239	0.04	*Annual Review of Entomology*
12	218	0.05	*Journal of Food Science*
13	201	0.04	*Food Engineering Reviews*
14	182	0.01	*Food Control*
15	174	0.02	*International Journal of Food Microbiology*

**Table 2:** Author information table of the top 20 published articles.

Anker	Count	Centrality	Author	Anker	Count	Centrality	Author
1	40	0.05	Baributsa D	11	16	0.05	Baoua IB
2	33	0.02	Murdock LL	12	15	0.00	Du SS
3	27	0.00	Arthur FH	13	15	0.01	Opit GP
4	23	0.00	Jayas DS	14	15	0.01	Stejskal V
5	23	0.02	Liu ZL	15	14	0.00	Coradi PC
6	20	0.00	White NDG	16	14	0.01	Daglish GJ
7	19	0.00	Athanassiou CG	17	14	0.00	Hubert J
8	18	0.01	Mvumi BM	18	13	0.00	Amadou L
9	17	0.01	Elias MC	19	13	0.01	De Oliveira M
10	17	0.01	Maier DE	20	13	0.01	Liu QZ

**Table 3:** Information table of the top 20 major research countries with the published article.

Anker	Count	Centrality	Country	Anker	Count	Centrality	Country
1	354	0.17	USA	11	39	0.03	ITALA
2	243	0.10	CHINA	12	38	0.03	KENYA
3	173	0.08	BRAZIL	13	36	0.00	MEXICO
4	126	0.11	CANADA	14	35	0.02	ARGENTINA
5	78	0.06	AUSTRALIA	15	34	0.04	NIGERIA
6	69	0.07	INDIA	16	34	0.05	GREECE
7	59	0.02	PAKISTAN	17	31	0.07	SPAIN
8	54	0.3	ENGLAND	18	27	0.04	JAPAN
9	42	0.02	KOREA	19	26	0.00	POLAND
10	42	0.04	GERMANY	20	25	0.00	EGYPT

**Table 4:** Information table of the top 20 major research institutions with published articles.

Anker	Count	Centrality	Institution
1	86	0.23	Purdue University
2	55	0.17	Kansas State University
3	51	0.14	Agricultural Research Institute
4	41	0.08	USDA Agricultural Research Service
5	39	0.05	China Agricultural University
6	36	0.08	Federal University of Vicosa
7	36	0.06	University of Manitoba
8	29	0.08	Contact Agriculture and Agri-Food Canada
9	27	0.12	University of Agriculture Faisalabad
10	26	0.02	Oklahoma State University
11	22	0.02	University of Thessaly
12	22	0.00	University of Arkansas
13	22	0.09	Iowa State University
14	21	0.07	Univ Fed Pelotas Univ Fed Pelotas
15	20	0.03	University of Zimbabwe
16	20	0.08	International Maize and Wheat Improvement Center CIMMYT
17	20	0.01	Beijing Normal University
18	19	0.01	Federal University of Santa Maria
19	18	0.04	Henan University of Science and Technology
20	17	0.01	Chinese Academy of Agricultural Sciences

**Table 5:** Information table of the top 20 keywords.

Ranker	Count	Centrality	Keyword
1	196	0.10	grain
2	191	0.07	storage
3	166	0.16	temperature
4	166	0.07	coleoptera
5	128	0.08	quality
6	119	0.04	wheat
7	108	0.04	tribolium castaneum
8	84	0.05	essential oil
9	80	0.04	sitophilus zeamai
10	76	0.06	resistance
11	69	0.04	rhyzopertha dominica
12	68	0.06	growth
13	66	0.08	carbon dioxide
14	65	0.04	maize
15	64	0.02	toxicity
16	64	0.02	hermetic storage
17	61	0.09	insect
18	59	0.01	sitophilus oryzae
19	59	0.05	protein
20	55	0.02	pest

## Data Availability

Data is contained within the article.
